# Genomic Prediction for Growth‐Related Traits in Golden Pompano ( *Trachinotus ovatus* )

**DOI:** 10.1111/eva.70147

**Published:** 2025-08-26

**Authors:** Huibang Sun, Miaomiao Zheng, Cun Wei, Quanqi Zhang, Jinxiang Liu

**Affiliations:** ^1^ MOE Key Laboratory of Marine Genetics and Breeding, College of Marine Life Sciences/Key Laboratory of Tropical Aquatic Germplasm of Hainan Province, Sanya Oceanographic Institution Ocean University of China Sanya China; ^2^ Laboratory for Marine Fisheries Science and Food Production Processes, Qingdao Marine Science and Technology Center Qingdao China; ^3^ Hainan Seed Industry Laboratory Sanya China; ^4^ Shandong Key Laboratory of Marine Seed Industry (Preparatory) Ocean University of China Qingdao China

**Keywords:** genomic prediction, golden pompano, growth traits, whole‐genome resequencing

## Abstract

Golden pompano ( *Trachinotus ovatus* ) is a rapidly growing marine aquaculture species along the southeast coast of China due to its favorable biological traits. However, the relatively short domestication history of marine species compared to terrestrial livestock and crops indicates untapped genetic potential. Therefore, selective breeding in marine aquaculture presents a significant opportunity for genetic improvement. This study aimed to establish a comprehensive genomic prediction to support the selection of new fast‐growing varieties of golden pompano. Body weight was selected as the primary trait for evaluating growth traits. Whole‐genome sequencing was performed on 692 samples, resulting in 4,886,850 high‐quality SNPs after filtering. Three SNP selection strategies were used for evaluating the genomic prediction accuracy, including the Evenly method, GWAS‐based method, and Random method. We addressed the issue of overestimation in the GWAS‐based method. After implementing cross‐validation, the GWAS‐based method demonstrated superior predictive accuracy across most SNP sets. Additionally, six breeding models were evaluated for their performance in genomic prediction, with GBLUP showing higher predictive ability. In terms of SNP density, we determined that 5000 SNPs selected via the Evenly method and 7000 SNPs selected via the GWAS‐based method represent optimal densities for accurately predicting body weight in golden pompano. These findings provide valuable insights for reducing breeding costs while improving selection accuracy, providing a practical strategy for the selection of golden pompano with economically valuable growth traits in aquaculture breeding programs.

## 1 Introduction

Golden pompano is a warm‐water pelagic fish that inhabits tropical, subtropical, and temperate marine environments. It possesses desirable biological characteristics, including a rapid growth rate, high levels of unsaturated fatty acids, and a strong adaptability to marine cage culture. Therefore, golden pompano has long been regarded as an economically significant marine cultured species. However, the rapid expansion of large‐scale aquaculture, coupled with a lack of effective management and selective breeding of parental stocks, has resulted in diminishing within golden pompano populations. This decline is reflected in slower growth rates, lower egg hatching rates, and increased susceptibility to disease among the fish. Currently, the golden pompano breeding program primarily relies on annual phenotypic selection, focusing mainly on traits such as weight. This presents a significant opportunity for advancements through molecular breeding.

Genomic selection (GS) is a marker‐assisted selection method that utilizes high‐density molecular markers across the whole genome to enhance selective breeding (Meuwissen et al. 2001). To date, GS has been widely applied to improve the phenotypes of various economically important aquatic species, including fish, shellfish, and shrimp (Dong et al. 2016; Luo et al. 2022; Wang et al. 2018). Compared to traditional breeding methods, GS offers several advantages. It enhances breeding efficiency and shortens the breeding cycle by enabling early selection of candidates based solely on genomic estimated breeding values (GEBVs), without the need for phenotypic records (Hayes et al. 2009). This is particularly useful for traits that are difficult or time‐consuming to measure and allows faster genetic gain per unit of time. It improves the accuracy of breeding value predictions by associating phenotype and genotype data, especially in low‐heritability traits (Goddard and Hayes 2009). GS can mitigate the potential limiting effects of genotype by environment (G × E) interactions by enabling more accurate predictions of breeding values across diverse environmental conditions (Mulder 2016). Meanwhile, one of the main advantages of genomic selection in aquaculture is its ability to enable within‐family selection for traits that cannot be measured directly on selection candidates, such as carcass composition or resistance to specific pathogens. Ultimately, GS is anticipated to decrease inbreeding while increasing the rate of genetic gain at a given level of inbreeding (de Roos et al. 2011; Scott et al. 2021). GS has been widely employed as an effective tool for genetic improvement due to its advantages in shortening the breeding cycle and enhancing genetic prediction accuracy.

The core issue of GS is the accurate estimation of GEBV. The effect of GS depends on the accuracy of genomic prediction (GP). Typically, the GP is derived from the correlation between the GEBV and true breeding values. The GP accuracy is influenced by different factors, including the size of the genome, heritability of traits, reference population size, SNP density, the method of estimation, and the relationship between the reference and candidate population (Daetwyler et al. 2010; Gao et al. 2012; Lund et al. 2011; Pszczola et al. 2012). Theoretically, the accuracy of GP improves with the increased SNP density. In fact, the increase in marker density might induce noise makers and high costs. The strategies for reducing SNP density to obtain high accuracy have been reported in aquaculture species, and the predictive ability was found to be effective (Gong et al. 2022; Robledo et al. 2018; Song and Hu 2022). Additionally, the methods for obtaining SNP sets and breeding models also influenced the prediction accuracy (Allal and Nguyen 2022). It is essential to estimate predictive ability according to the desired scenario. To date, the studies on performing accuracy evaluation of GP have been limited. Understanding the optimal design for the required SNP density and breeding model is beneficial and to be expected from the implementation of GS. It is significant to assess the potential of GS for growth traits in golden pompano. In this study, we integrate our previous GWAS results on growth traits in golden pompano with newly conducted GP using the data from a breeding population of 692 individuals. We evaluated the prospects for genetic prediction of growth traits, focusing on SNP selection methods and predictive models. By establishing a comprehensive evaluation system for GP accuracy of economically important traits in golden pompano, this study contributed valuable insights toward developing practical and cost‐effective GS strategies for aquaculture.

## 2 Material and Methods

### 2.1 Sample Collection

The samples were collected from Hainan Chenhai Aquatic Co. Ltd. which was described in Zhu et al. (Zhu et al. 2023). At the age of 10 months, a total of 692 individuals farmed in different marine cages were stochastically randomly collected, and growth‐related traits, including body weight (BW) and body length (BL), were measured for further study. The muscle tissue sampled from the back of golden pompano was immediately stored at −80°C for subsequent DNA extraction.

### 2.2 Genotyping, Quality Control, and GWAS

The raw reads for each sample were quality controlled using Fastp before SNP calling, removing the reads with low‐quality bases, unidentified nucleotides, and barcode adaptor. Subsequently, the filtered clean reads aligned to the assembled golden pompano genome (Zhang et al. 2019) using the BWA program with default parameters (Li and Durbin 2010). Following this alignment, the data were utilized for genotyping, and all indels and nonbiallelic SNPs were removed. Quality control for SNP data was conducted using PLINK1.9 for the subsequent association analysis (Purcell et al. 2007). The filtering parameters were summarized as follows: (1) individuals with an SNP marker call rate < 0.9; (2) genotype call rate < 0.9; (3) minor allele frequency (MAF) < 0.05. SNP annotation was performed using SnpEff software (Cingolani et al. 2012). All missing SNPs were imputed with Beagle v5.4 software (Browning and Browning 2007). To identify SNPs associated with body weight, GWAS analysis was performed using the GEMMA software, following the mixed linear model (MLM) implemented in our previous study (Zhu et al. 2023). The following model was applied:

y=Xb+Zu+e

where *y* represents the vector of observed phenotypes (body weight); *b* is the vector of fixed effects; *X* and *Z* are the incidence matrices for fixed effects and genetic effects, respectively; *u* is the vector of random additive effects, following a normal distribution *N* (0, G $\sigma_{g}^{2}$), where *G* is the genomic relationship matrix and $\sigma_{g}^{2}$ is the additive genetic variance, and *e* is the vector for residual error with the distribution *N* (0, I $\sigma_{e}^{2}$), where *I* is an identity matrix and $\sigma_{e}^{2}$ represents residual variance (VanRaden 2008).

### 2.3 Obtaining SNPs by Different Selection Strategy

The SNP selection strategy plays a critical role in the calculation of breeding values, as it can significantly influence the accuracy of phenotype prediction. According to our previous study (Zhu et al. 2023), we found that the gene loci used in this study exhibited strong linkage, suggesting that the Evenly method may provide more stable predictions. Given the absence of pedigree data, we used the Evenly method as a criterion to evaluate the accuracy of other SNP selection strategies. We employed three SNP selection strategies in this study: (1) GWAS‐based method: SNPs were selected based on the results of a single‐SNP GWAS analysis performed using GEMMA to identify SNPs associated with body weight. SNPs were ranked in ascending order according to their *p* values from the GWAS analysis. (2) Evenly method: SNPs were evenly extracted from all quality‐control‐passed SNPs at fixed intervals. The chromosomes were divided into segments corresponding to the required number of SNPs, and the midpoint SNP from each segment was selected. (3) Random method: SNPs were randomly selected across the genome. The names of all SNPs in the VCF file were extracted, and the required number of SNPs were randomly extracted using the sample function of the R package. We compared the accuracy of phenotypic predictions across the different SNP selection strategies to determine the optimal approach. The distribution of SNPs (10,000), based on different selection strategies, was visualized using the CMplot R package (Yin et al. 2021).

### 2.4 Genomic Prediction Models

The selected SNPs were used as predictors of the phenotype or GEBV for the body weight trait of golden pompano. All Bayes models were fitted to the data using BGLR/R (Pérez and de los Campos 2014), which supports Bayesian regression models, including BayesA (BA), BayesB (BB), BayesC (BC), Bayes LASSO (BL), and Bayesian Ridge Regression (BRR). These Bayesian models are well‐suited for genomic selection due to their ability to handle the “large p, small n” problem. For models implemented in BGLR, we used the default prior distributions and 10,000 MCMC iterations with a burn‐in of 2000. Genomic data often involve more markers (features) than individuals (observations). Bayesian models naturally address this problem by imposing regularization through priors, reducing the risk of overfitting. GBLUP uses a linear mixed model, which is flexible and computationally efficient. This framework is ideal for simultaneously estimating breeding values and accounting for environmental effects. Meanwhile, GBLUP is based on the infinitesimal model, which assumes that many loci contribute small effects to a trait. This aligns well with the genetic architecture of many complex traits (such as growth trait) (VanRaden 2008). Markers were treated as random effects, capturing the majority of the genetic variance. The model generally follows this form:

y=μ+Xβ+e

where *y* is a vector representing estimated breeding values (EBV) or, for continuous phenotypes (such as body weight trait), a vector of liabilities. *μ* is a vector of fixed effects (intercept only), *β* is the vector of marker effects treated as random, and *X* is an *n*x*p* matrix of SNP genotypes, with *n* being the number of individuals and *p* the number of markers. Genotypes are encoded based on the count of the minor allele (0, 1, or 2). The vector *β* (*p*x1) comprises the SNP effect coefficients to be estimated. Lastly, *e* represents random residuals, which are assumed to follow a normal distribution: e ∼ N (0, I $\sigma_{e}^{2}$). Here, *I* is an identity matrix of appropriate dimensions, and $\sigma_{e}^{2}$ is the residual variance.

The GBLUP model assumes equal variance for all markers and estimates the genomic relationship matrix based on genome‐wide SNPs.

Bayesian models differ in their prior assumptions on marker effects:

- BayesA uses a scaled‐t prior allowing for a heavy‐tailed effect size distribution.
- BayesB assumes that a proportion of SNPs have no effect (spike‐and‐slab prior).
- BayesC assigns a common variance to all nonzero effect markers, with a fixed proportion of SNPs having zero effect.
- Bayes LASSO applies a double‐exponential (Laplace) prior, promoting sparse solutions.
- Bayesian Ridge Regression applies Gaussian priors (L2 penalty) similar to ridge regression.

### 2.5 Evaluation of Genomic Prediction Accuracy at Varying SNP Marker Densities

To assess the impact of SNP marker density on GP accuracy, we conducted a series of analyses using SNP sets of varying densities. For this study, SNP datasets were generated at several density levels, ranging from low (100 SNPs) to high (10,000 SNPs), allowing for a comprehensive evaluation of predictive performance. The SNP datasets consisted of seven subsets with relatively large gaps between SNP counts. However, determining the optimal SNP selection method and the most suitable breeding model requires careful consideration of multiple factors. To fine‐tune the selection process and narrow down the optimal number of SNPs, we further refined the range of variation between the SNP subsets. Each subset underwent the construction of breeding models, including BA, BB, BC, BL, BRR, and GBLUP, and the predictability for each was assessed through five‐fold cross‐validation, ensuring robust evaluation across different data partitions.

### 2.6 Genomic Prediction and Cross‐Validation

The GP accuracy was estimated using five‐fold cross‐validation, with each cross‐validation repeated five times. In this process, the population was randomly divided into five equal subsets. For each iteration, one subset was designated as the validation set (20% of the data), while the remaining four subsets (80% of the data) served as the training set. This procedure was repeated five times; a different subset was used as the validation set in each iteration, ensuring stable results.

For each training set, SNP discovery was conducted using the GWAS result in the training population for the GWAS‐based SNP selected method. The phenotypes in the validation set were masked, and GP accuracy was estimated using various breeding models, including BA, BB, BC, BL, BRR, and GBLUP. The training population was used for GWAS analysis, model construction, and estimation of marker effects, which were then applied to calculate the accuracy of GP of individuals in the validation set. The accuracy of each model was evaluated by comparing the correlation between predicted GEBVs and the masked phenotypes in the validation population. To ensure consistency, the same training and validation sets were used across all models for each cross‐validation loop. The accuracy of GP was primarily determined by the correlation coefficient between the GEBVs and observed phenotypes.

### 2.7 Assessment of Reference Population Size on GP Accuracy

To evaluate the impact of training population size on genomic prediction (GP) accuracy, we constructed five subsets of the training population consisting of 100, 200, 300, 400, and 500 individuals, respectively, from a reference group of 554 fish. The same validation population of 138 individuals was used throughout to ensure comparability across scenarios. Three SNP selection strategies were tested under each training size condition: (1) GWAS‐based selection (top 10,000 SNPs ranked by −log_10_(*p*) value); (2) Random selection of 10,000 SNPs; (3) Evenly selected 10,000 SNPs across the genome. For each combination of training size and SNP selection strategy, five independent replicates were generated through random sampling. The GBLUP model was used to estimate genomic breeding values, and the Pearson correlation between observed and predicted phenotypes in the validation set was used to assess prediction accuracy.

## 3 Results

### 3.1 Data Filtering, Phenotypic Statistics, and Phylogenetic Analysis

**FIGURE 1:**
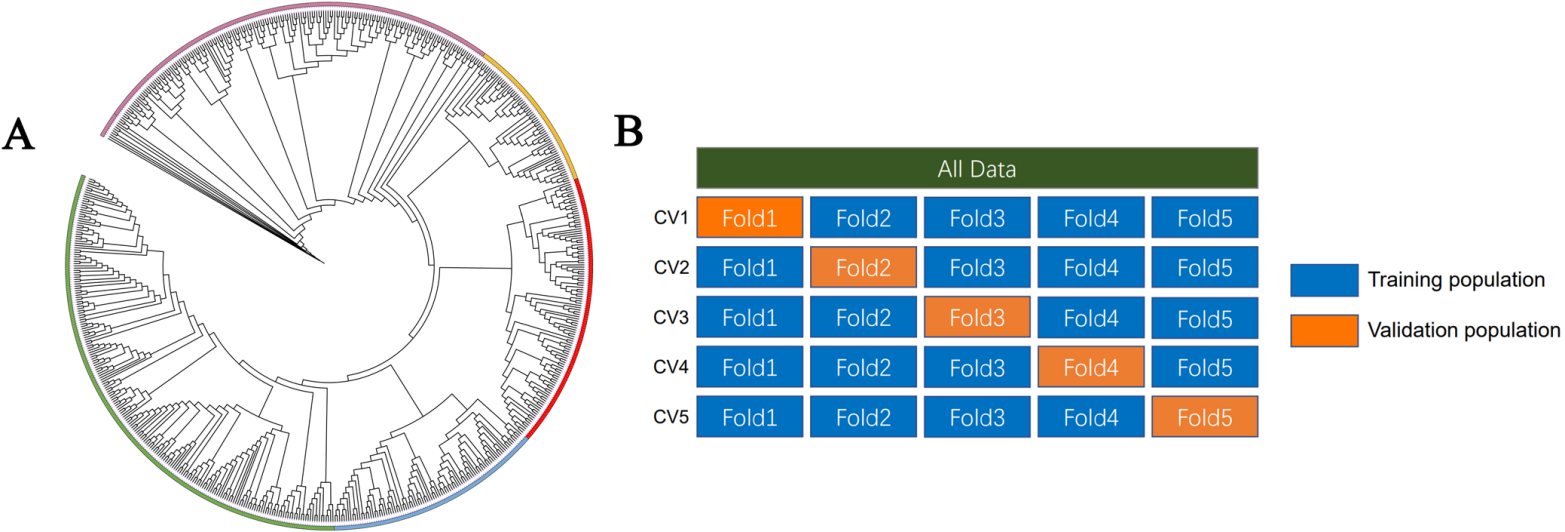
(A) A phylogenetic tree was constructed based on genome‐wide SNPs to evaluate the genetic relationships among the 692 golden pompano individuals. (B) A schematic diagram illustrating how the population is divided in a five‐fold cross‐validation framework. Abbreviation: CV: Cross‐validation.

Through the whole genome resequencing, a total of 3.4 Tb raw data was generated, with an average sequencing depth was 6 × and an average genome coverage of 85%. Following the filtration of individuals and SNPs with high missing rates and low quality, we obtained a total of 4,886,850 QC‐filtered SNPs from 692 samples. According to our research, the mean and standard deviation values for the growth‐related traits (BW) were 655.64 ± 213.15 g. As shown in Figure 1A, the phylogenetic tree constructed from genome‐wide SNP data revealed that the 692 golden pompano individuals could be grouped into five major genetic clusters, suggesting clear population differentiation. Given the observed population structure, we applied five‐fold cross‐validation to estimate the genomic prediction accuracy in a robust and unbiased manner (Figure 1B).

### 3.2 Effects of SNP Selection Strategies on Genomic Prediction Accuracy

**FIGURE 2:**
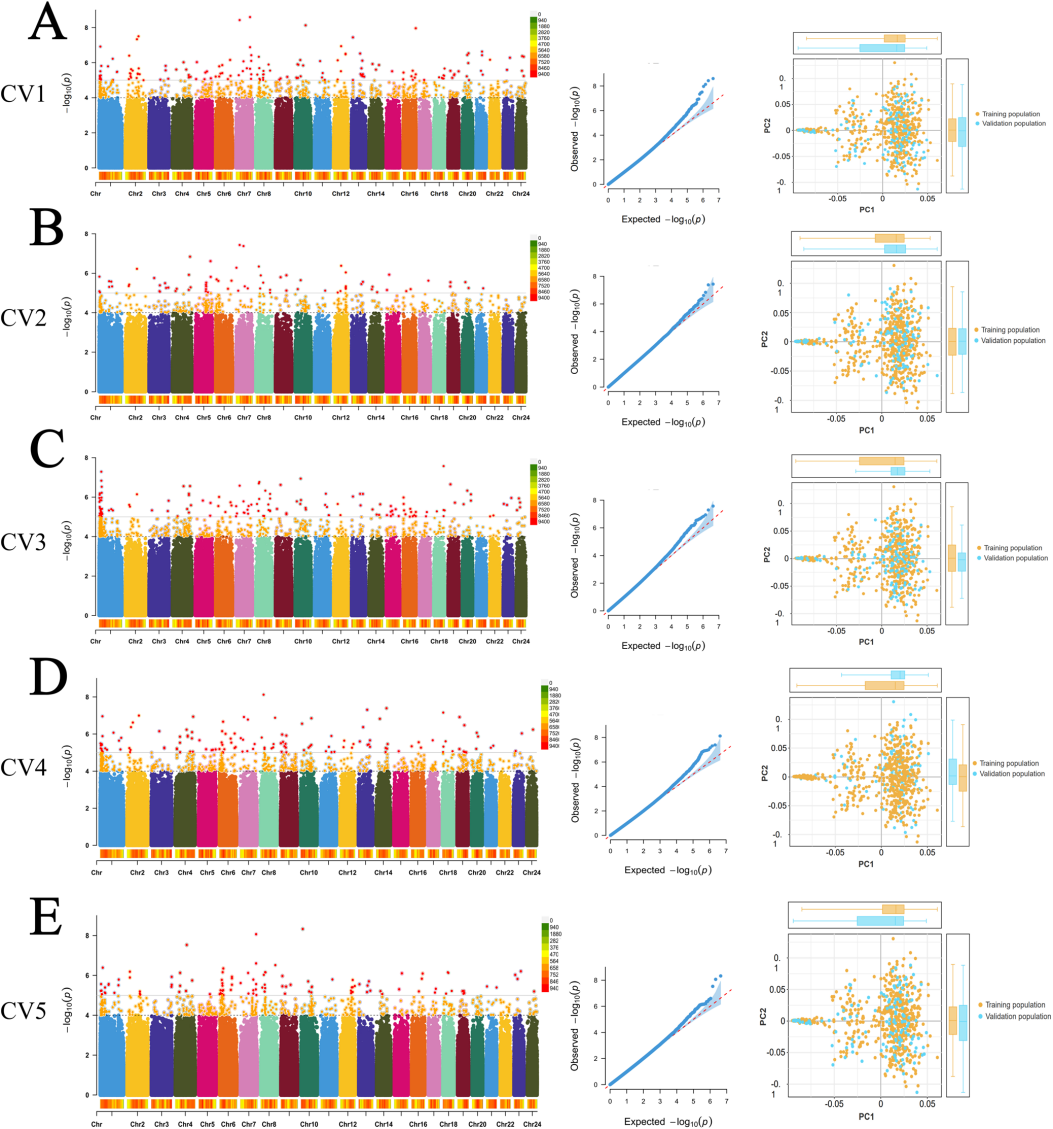
Manhattan plot, QQ plot, and PCA plot derived from the cross‐validation process of the GWAS results in Golden pompano. The figures in each row, arranged sequentially from left to right, represent the Manhattan plot, QQ plot, and PCA plot. In Manhattan plot, each dot represents a single SNP, plotted according to its genomic position (x‐axis) and the –log_10_(*p* value) of its association with the trait (y‐axis). Yellow points represent SNPs with –log_10_(*p* value) values between 4 and 5, and red points represent those exceeding 5. In PCA, each point represents an individual, projected onto the first two principal components (PC1 and PC2). Colors indicate different groups (training population and validation). Boxplots along the x‐ and y‐axes illustrate the distributions of PC1 and PC2 for each group, helping to visualize population separation in cross‐validation.

**FIGURE 3:**
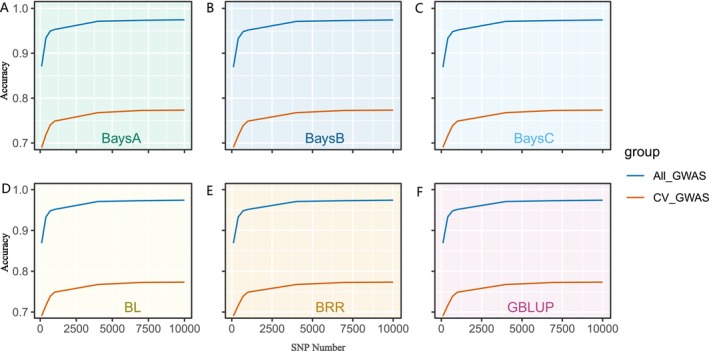
Comparison of predictive accuracies using different selection strategies during GWAS‐based selection for body weight trait. (A–E) The predictive accuracies by BA, BB, BC, BL, BRR, and GBLUP, respectively. The different models and SNP densities were used for the analysis. The x‐axis represents different SNP numbers. The y‐axis represents the correlation coefficient between GEBV and real phenotypes.

Before proceeding with GP estimation, we first compared the predictive accuracy of SNP selection methods based on GWAS conducted in different populations (only training population or all population) (Figure 2). The results demonstrated clear differences in predictive accuracy depending on whether GWAS analysis was performed in the training population (CV_GWAS) (Figure 2A–E) or the entire population (All_GWAS). Specifically, the GP accuracy across different SNP sets was consistently high when the SNPs were selected using the All_GWAS strategy, with predictive reliability exceeding 0.85 for each SNP set (Figure 3). However, upon comparing the results of the All_GWAS strategy with those of the CV_GWAS approach, we observed that the predictive accuracy of the All_GWAS method may have been significantly overestimated. The CV_GWAS method, which selects SNPs only from the training population, provides a more realistic and less biased estimate of GP accuracy. In the subsequent analysis, the CV_GWAS method was employed for estimating genomic GP accuracy.

**FIGURE 4:**
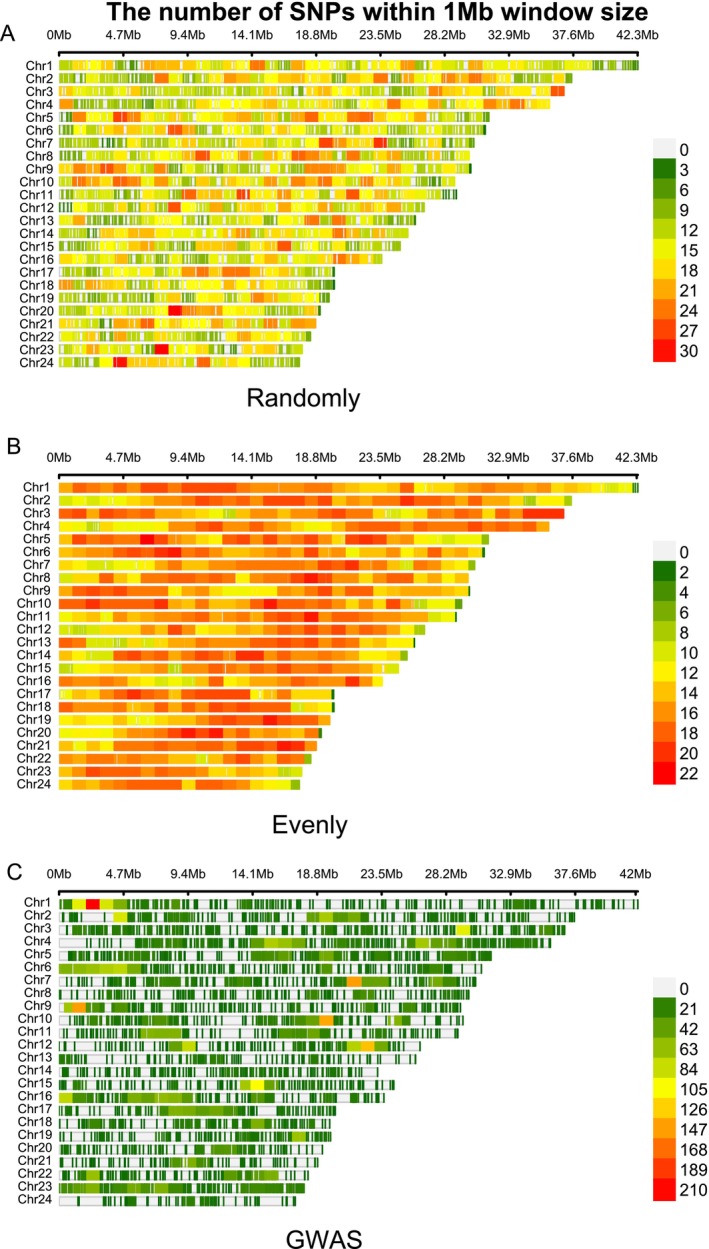
Density and distribution of the high‐quality SNPs (1000) based on different SNP selection methods. (A) SNPs density and distribution obtained by Random selection. (B) SNPs density and distribution obtained by Evenly selection. (C) SNPs density and distribution obtained by GWAS‐based selection.

**FIGURE 5:**
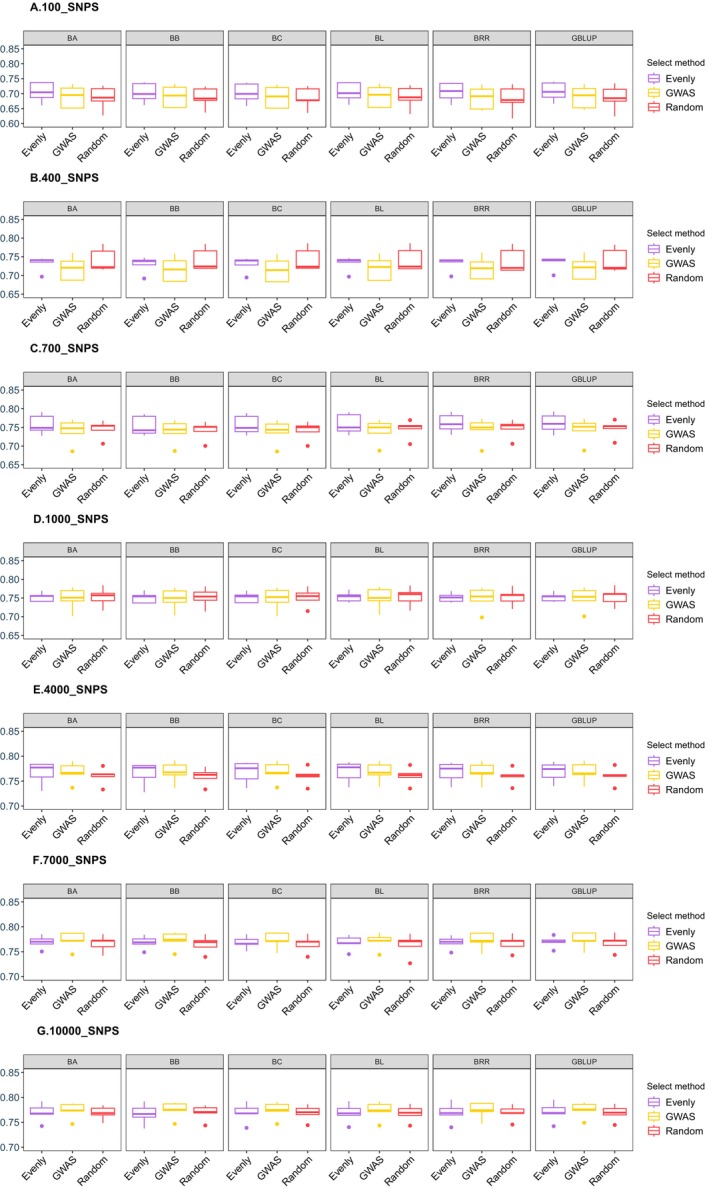
Genomic predictability for growth traits with different models, SNP density, and SNP selection methods in golden pompano. (A–G) The GP accuracy in different models and SNP densities from 100 to 10,000. (A) 100 SNPs; (B) 400 SNPs; (C) 700 SNPs; (D) 1000 SNPs; (E) 4000 SNPs; (F) 7000 SNPs; (G) 10,000 SNPs.

**FIGURE 6:**
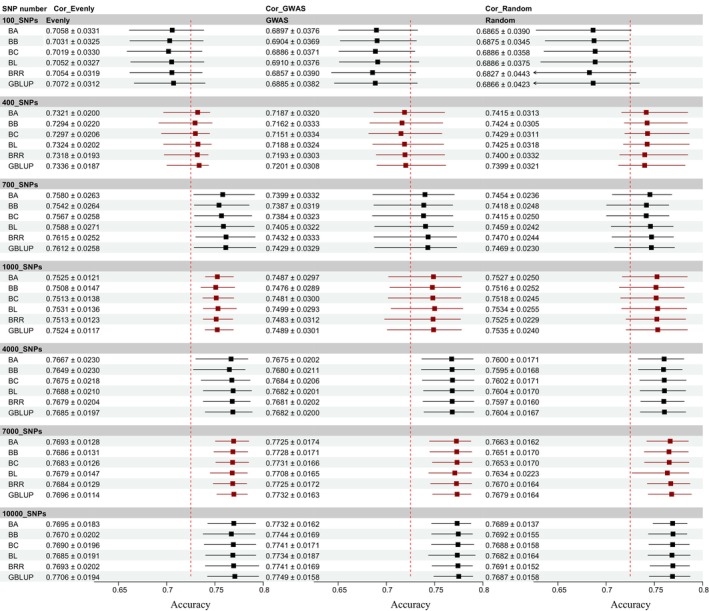
The forest plot of GP accuracy for body weight with different breeding models. The x‐axis represents the correlation coefficient between GEBV and real phenotypes. Cor_Evenly represents the correlation coefficient (average ± std. for result of five‐fold cross‐validation) with the Evenly SNPs select method. Cor_GWAS represents the correlation coefficient (average ± std. for result of five‐fold cross‐validation) with the GWAS SNP select method. Cor_Random represents the correlation coefficient (average ± std. for result of five‐fold cross‐validation) with the Random SNP select method.

Theoretically, all the SNPs should be selected for GP in GS. However, in practical applications, the inclusion of excessive or invalid SNPs can dilute the genetic signal and reduce the accuracy of GP. Usually, the SNP selection strategies significantly influence the accuracy of GP, given that the different SNPs may associate with varying genetic effects on the trait of interest. To estimate the impact of SNP selection on GP accuracy, we compared three SNP selection strategies—GWAS‐based, Random, and Evenly methods—under different models and across varying SNP densities. As shown in Figure 4, the distribution of SNPs differs significantly across selection strategies, influencing the coverage and localization of genetic markers across the genome. This has a pronounced effect on the accuracy of phenotype prediction. Our results revealed substantial differences in GP accuracy across the SNP selection strategies, with the accuracy improving as the SNP panel size increased. Specifically, under a high‐density SNP panel (SNPs number ≥ 7000), the GWAS‐based method (accuracy > 0.77) outperformed the other methods (accuracy < 0.77), delivering consistently higher predictive accuracy (Figures 5 and 6). These results highlight the importance of the SNP selection strategy in GS applications to optimize predictive performance, depending on the available SNP density. The GP accuracy for different SNP selection strategies is documented in Table S1.

### 3.3 Predictability of GBLUP and Bayesian Models

In this study, we evaluated the predictability of GBLUP and five Bayesian methods using seven subsets of SNP data. The analysis revealed consistent patterns in the predictive abilities of different breeding models, with significant variations in GP accuracy (Figure 6). Interestingly, each model exhibited distinct characteristics depending on the SNP selection strategies. Under the GWAS‐based and Random SNP selection methods, although the variation in accuracy was relatively smooth, the correlation coefficient between GEBV and actual phenotypes increased with the number of SNP densities (Figure 6). For the Evenly method, the overall correlation coefficient improved, but a drop was observed in the 1000 SNP set (Figure 6). Meanwhile, the GP accuracy under the GWAS‐based method (~0.775) was slightly higher than that of the Evenly method (~0.770) and Random method (~0.769), improving about 6%. These findings suggest that data derived from the SNP selection strategy GWAS‐based method are more effective in evaluating the prediction accuracy of different models.

As shown in Figure 6, the modals of BL, BRR, and GBLUP demonstrated slightly better predictive accuracy for growth traits GEBV of golden pompano in all SNP sets under the Evenly method. For the GWAS‐based method, BRR and GBLUP performed better, while BA and GBLUP showed superior performance under the Random selection method. Across all SNP selection strategies, GBLUP consistently emerged as the best‐performing model in terms of correlation coefficient under each SNP panel density. Therefore, we selected GBLUP for subsequent evaluations of the impact of SNP density on GP accuracy. The GP accuracy for GBLUP and Bayesian models is documented in Table S2.

### 3.4 Picking the Optimal Number of SNPs

**FIGURE 7:**
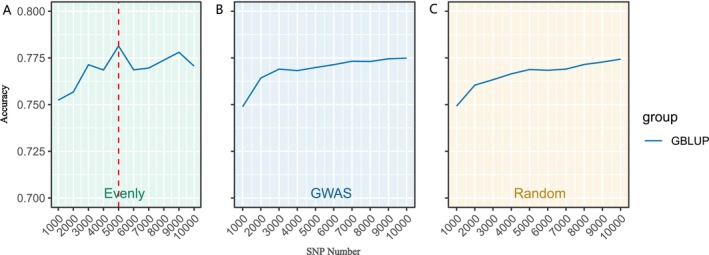
The GP accuracy at different SNP densities and SNP selection strategies. (A) The GP accuracy at different SNP densities with Evenly method. (B) The GP accuracy at different SNP densities with GWAS‐based method. (C) The GP accuracy at different SNP densities with Random method. X‐axis represents different SNP numbers. Y‐axis represents the correlation coefficient between GEBV and real phenotypes.

Analyzing the outcomes presented in Figure 5 and Figure 6, it became clear that SNP panels ranging from 1000 to 10,000 played a crucial role in improving the accuracy of GP. To refine the selection of SNPs and pinpoint the optimal SNP panel for GP, further analysis was performed to improve precision in prediction. Based on the performance of breeding models and SNP selection strategies, GBLUP was selected to evaluate the influence of different SNP panel densities on GP accuracy. The results illustrated a significant improvement in predictive accuracy with SNP panel size increased (Figure 7). However, the trends varied across different SNP selection strategies. For Evenly method, the accuracy did not consistently increase with a larger SNP panel. Instead, it peaked at around 5000 SNPs, after which no further improvements were observed (Figure 7A). For GWAS‐based and Random methods, the accuracy consistently increased with the number of SNP density growth. A substantial improvement was observed as the SNP panel reached 7000 sites, and the accuracy nearly plateaued by 9000 SNPs, where it reached its maximum in the GWAS‐based method (Figure 7B). For the Random selection method, the trend in accuracy was the same as that of the GWAS‐based method, but the overall accuracy was slightly lower at each SNP panel size. This suggested that a SNP panel of 9000 markers represents the optimal number for achieving high GP accuracy in golden pompano, particularly when using the GWAS‐based method. However, from an economic perspective, a SNP panel of 7000 markers may be considered a cost‐effective alternative, as it still delivers reliable prediction accuracy with only a slight reduction compared to the 9000 SNP panel. This balance between accuracy and cost could be advantageous for breeding programs. The GP accuracy across varying numbers of SNPs is documented in Table S3.

### 3.5 Effective of Reference Population Size on GP Accuracy

**FIGURE 8:**
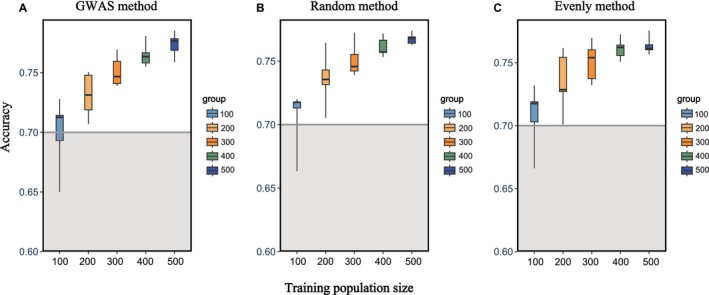
The GP accuracy at different training populations. X‐axes illustrate the number of training populations. Y‐axes illustrate the GP accuracy. (A) Effect of training population size on GP accuracy with GWAS‐based SNP selection method. (B) Effect of training population size on GP accuracy with random SNP selection method. (C) Effect of training population size on GP accuracy with evenly SNP selection method.

As shown in Figure 8, GP accuracy generally improved with increasing training population size across all three SNP selection strategies. However, clear differences were observed in how each method responded to changes in training population size. The GWAS‐based method showed the lowest accuracy among the three strategies when the training population size was ≤ 400, indicating its higher sensitivity to training sample size. For example, with 100 individuals, the accuracy of the GWAS‐based method was approximately 0.700, while the random and evenly distributed methods reached 0.706 and 0.707, respectively. Moreover, the range of prediction accuracy across training population sizes further highlighted the sensitivity of each method. The GWAS and Random methods had the largest fluctuations (0.700–0.774 and 0.706–0.774, respectively), while the evenly distributed method had the narrowest range (0.707–0.764), indicating that it was the least affected by changes in training population size. The GP accuracy at varying reference population sizes is documented in Table S4.

## 4 Discussion

The predictive capability of GS relies on the availability and quality of molecular markers. In theory, a higher number of markers improves the prediction ability. However, in practical applications, the choice of genotyping technology plays a critical role in marker acquisition, and each technology processes distinct advantages and limitations. Common methods include RAD sequencing, SNP chip arrays, and whole‐genome resequencing, each differing in the number of markers they generate, the costs involved, and the overall resolution. Moreover, the prediction accuracy was significantly different between species and traits of interest, such as in Pacific white shrimp (Wang et al. 2017), large yellow croaker (Dong et al. 2016), Nile tilapia (Yoshida et al. 2019), and Atlantic salmon (Tsai et al. 2015). In these studies, the GP accuracy was influenced by a number of factors, including the breeding models, SNP panel density, marker selection strategy, and the effective population size. Therefore, a detailed assessment of these factors is essential for more accurate predictions and the effective implementation of GS strategies. The most effective strategy requires a careful balance between genotyping technology, marker density, and economic constraints. A thorough evaluation of these factors is crucial for maximizing the effectiveness of GS and achieving higher rates of genetic gain in breeding programs. Whole‐genome resequencing is, to date, arguably the most powerful genotyping method. Compared with other genotyping techniques, it enables the identification of a much larger number of SNPs.

The SNP selection strategy plays a crucial role in GP accuracy. In our study, three methods were employed for estimation in golden pompano growth trait, including GWAS‐based, Evenly, and Random methods. The GWAS‐based method showed the higher accuracy (~0.77) in GP compared with other methods. In a previous study, the GWAS‐based method could also increase the accuracy (~9.2%) of GP in Russian sturgeon body weight trait (Song et al. 2024). In the growth traits GS study of Rock Bream, the SNP selected method based on GWAS results also demonstrated superior prediction accuracy (Gong et al. 2022). In GS, it could improve the GP accuracy according to adding the preselected SNP markers obtained from GWAS analysis in traits of growth under chronic thermal stress in rainbow trout, *Edwardsiella tarda* resistance in Japanese flounder, and intra muscular fat in Australian sheep (Lu et al. 2020; Moghaddar et al. 2019; Yoshida and Yáñez 2022). In the current study, we first estimated the GWAS‐based method from the training population (CV_GWAS) or from the entire population (All_GWAS). The results indicated that the predictive accuracy of the All_GWAS method was significantly overestimated (> 0.95), but the CV_GWAS method was a realistic and less biased estimate of GP accuracy. The overestimated predictive accuracy (> 0.95) was displayed in a previous GS study of orange‐spotted grouper (Shan et al. 2021; Shan et al. 2023). The higher reliability observed in the All_GWAS strategy could likely be attributed to the overlap between the discovery and validation populations, which might have introduced bias into the model. This overlap could artificially inflate accuracy estimates, as the SNPs identified from the whole population were optimized to maximize the prediction accuracy for the given dataset. According to our conjecture, we hypothesized that this was particularly evident in aquatic species due to the relatively short domestication history of their breeding population, leading to an LD distance and the genetic structure toward the wild population. To avoid overestimating prediction accuracy, it was crucial to separate the populations into distinct training and validation populations when applying a GWAS‐based method. This separation ensured that the markers identified in the training population were evaluated independently in the validation population, providing a more unbiased and realistic estimation of their predictive capability. One limitation of our study is that both GWAS and genomic prediction analyses were conducted within the same dataset. In practice, the GWAS‐based method is often applied across cohorts (e.g., between year classes), where the linkage between selected SNPs and causative variants may differ due to recombination events or shifts in allele frequencies. Consequently, the predictive advantage observed in this study may be reduced in real‐world scenarios. As a future direction, we intend to apply the genomic prediction models developed here to the next‐generation population to evaluate their predictive accuracy and assess the model's transferability across generations. In addition, the evenly spaced SNP selection strategy relies on well‐annotated genome assemblies with accurate SNP coordinates, which may not be available for all species. These factors should be carefully considered when implementing SNP selection strategies in breeding programs, as they are crucial for evaluating the practical utility of genomic prediction models in real‐world settings.

In the GWAS‐based SNP selection strategy, SNPs are selected separately for each trait based on the *p* values obtained from individual trait‐specific GWAS analyses. When multiple traits are considered, this approach typically requires conducting separate GWAS for each trait, which can lead to the inclusion of a much larger number of SNPs for genomic prediction. This is in contrast to randomly or evenly selected SNP panels, which maintain a fixed number of markers regardless of the trait. Such trait‐specific selection substantially increases the cost of genotyping and sequencing in breeding programs, especially when dealing with multiple traits simultaneously. This limitation should be given more attention in future studies. To address the cost implications of multiple‐trait genomics selection, several strategies may be considered. One potential solution is to identify pleiotropic SNPs that are significantly associated with multiple traits, allowing for a reduced yet effective SNP set. Alternatively, multitrait GWAS models can be employed to jointly analyze correlated traits, enabling the selection of shared SNPs that contribute to overall genetic merit. Another promising approach is to apply dimensionality reduction techniques, such as principal component analysis (PCA) on phenotype matrices, followed by GWAS on the principal components. These methods can help reduce marker redundancy and improve cost‐efficiency without compromising prediction accuracy.

The calculation methods for GEBV were broadly classified into two categories, BLUP‐based models and Bayesian models. The GBLUP method calculates GEBV by substituting the traditional pedigree‐based relationship matrix (A) with a genomic relationship matrix (G) (VanRaden 2008). In contrast, Bayesian models rely on the variance distribution of SNP effects, accumulating the total variance to estimate GEBV (Habier et al. 2011). Generally, Bayesian approaches were better than GBLUP in GP accuracy for complex traits due to their ability to model more diverse genetic architectures. Previous studies have systematically evaluated the accuracy of these models under different traits, SNP density, and heritability. The results revealed that different species and traits often required specific breeding models for optimal accuracy, which has been observed in numerous species, including Atlantic salmon (Tsai et al. 2015), sea bream (Palaiokostas et al. 2016), rainbow trout (Vallejo et al. 2018), common carp (Palaiokostas, Kocour, et al. 2018), European sea bass (Palaiokostas, Cariou, et al. 2018), channel catfish (Garcia et al. 2018), and Nile tilapia (Yoshida et al. 2019). Across these studies, GS has consistently demonstrated superior predictive power compared to traditional pedigree‐based approaches. In our study, GBLUP emerged as the most suitable model for predicting growth traits in golden pompano, exhibiting high accuracy in GP. GBLUP is computationally efficient, robust to overfitting, and widely used due to its stability across different traits and population structures, particularly in aquaculture breeding programs involving species such as Atlantic salmon (Ajasa et al. 2024), common carp (Song and Hu 2022), and large yellow croaker (Dong et al. 2016). In these species, GBLUP has consistently demonstrated high stability and predictive accuracy. This observation is in agreement with the results of our study. A challenge in our study was the inability to directly compare our GS models with the traditional BLUP method based on pedigree data, due to incomplete pedigree information. This limitation in the process of comparing different models is the inability to compare the results of GS models with the traditional BLUP method based on pedigree data, due to incomplete pedigree information. It was also a common limitation in aquaculture compared to livestock and crop breeding. For golden pompano, our findings suggested that GBLUP offered a reliable model for growth traits, but for more complex traits or other aquaculture species, Bayesian approaches might be more suitable. Therefore, continuous refinement and trait‐specific evaluation were necessary to enhance the application of GS in aquaculture.

Usually, the SNP density is positively correlated with the accuracy of GP, particularly for quantitative traits such as growth traits, which are controlled by the polygenic effects of multiple genes. A higher number of SNP panels could improve the accuracy of GP by capturing more genetic variation across the genome within a specific scope. However, increasing the number of SNP markers also comes with higher genotyping costs and greater demands for computational resources. In aquaculture, where the economic value of individual organisms, such as fish, is relatively low compared to agricultural species like pig, cattle, or sheep, it is crucial to balance accuracy with cost‐efficiency. Identifying an optimal number of SNPs that maintain high predictive accuracy while reducing genotyping costs is of significant importance for aquaculture breeding programs. Our study highlighted the relationship between GP accuracy and SNP density, providing insights on selecting appropriate SNP densities to balance accuracy and economic returns. Previous studies have also demonstrated that optimal SNP densities are necessary for genomic prediction across species and traits. It was found that 2000 SNPs were sufficient to achieve similar accuracy to the full dataset when predicting resistance to amoebic gill disease in Atlantic salmon (Robledo et al. 2018). Similarly, in Pacific white shrimp, it was shown that a SNP density of 1000 was adequate for predicting tolerance to high salinity stress (Luo et al. 2022). For growth traits in Pacific oyster and Rock Bream, accurate predictions were achieved with 2500 and 1000 SNPs, respectively (Gong et al. 2022; Gutierrez et al. 2018). In this study, we determined the optimal SNP panel for growth traits in golden pompano by comparing the predictive accuracy of different SNP sets while confirming the best SNP selection method and genomic prediction model. Our results indicate that using 5000 SNPs selected by the Evenly method and 7000 SNPs from the GWAS‐based method provided near‐maximum GP accuracy, with little improvement observed beyond these thresholds. This suggests that 5000 (Evenly) and 7000 (GWAS‐based) SNPs represent optimal low‐density SNP panels for growth traits in golden pompano, offering a cost‐effective solution without compromising predictive accuracy.

Our findings reveal notable differences in the robustness of SNP selection strategies to variations in training population size. Although increasing the size of the training population generally enhances GP accuracy, the GWAS‐based SNP selection method exhibited the highest sensitivity to sample size, performing worst among the three methods at smaller training sizes (≤ 400). This could be attributed to the reduced statistical power of GWAS in small populations, leading to suboptimal SNP selection when sample sizes are limited. In contrast, the evenly distributed SNP selection method showed the most stable performance, with the smallest range of GP accuracy (0.707–0.764) across all training sizes. This suggests that distributing SNPs uniformly across the genome may buffer against sampling variation and provide more consistent prediction results, especially when the training dataset is small or moderately sized. These results underscore the importance of considering not only SNP informativeness but also robustness to training population size when selecting SNPs for genomic prediction, particularly in breeding programs where large reference populations may not always be feasible.

## 5 Conclusion

In summary, this study developed a comprehensive estimation for GP accuracy in golden pompano growth traits. Three critical components were evaluated at different levels, including SNP selection strategy, prediction models, and marker density. The results highlighted that the GWAS‐based SNP selection method consistently outperformed other methods in terms of predictive accuracy, especially when using a higher density of SNPs. Among the prediction models tested, GBLUP emerged as the most reliable model for predicting growth traits in golden pompano. Furthermore, an optimal SNP density was identified, with 5000 (Evenly method) and 7000 (GWAS method) SNPs providing the best balance between prediction accuracy and genotyping cost. These findings provide valuable insights into the effective application of GS in aquaculture, offering a balance between cost‐efficiency and predictive accuracy. This study also laid the foundation for future breeding programs aimed at improving economic traits in golden pompano.

## Consent

The authors have nothing to report.

## Conflicts of Interest

The authors declare no conflicts of interest.

## Supporting information


**Table S1.** Prediction accuracy results of three SNP selection methods.

**Table S2.** Prediction accuracy results of six breeding models.

**Table S3.** Prediction accuracy results of different SNP density.

**Table S4.** Prediction accuracy results of different training population sizes.

## Data Availability

The datasets of genotypes analyzed during the current study are available on figshare (https://doi.org/10.6084/m9.figshare.28217549.v1). The reference genome used is available on figshare (https://doi.org/10.6084/m9.figshare.7570727.v3). The phenotypic data is not publicly available since the populations are consisted of the nucleus herd of Hainan Chenhai Aquatic Co. Ltd, but are available from the corresponding author on reasonable request.
